# Recovery Education for Adults Transitioning From Homelessness: A Longitudinal Outcome Evaluation

**DOI:** 10.3389/fpsyt.2021.763396

**Published:** 2021-11-22

**Authors:** Anna Durbin, Rosane Nisenbaum, Ri Wang, Stephen W. Hwang, Nicole Kozloff, Vicky Stergiopoulos

**Affiliations:** ^1^MAP Centre for Urban Health Solutions, Li Ka Shing Knowledge Institute, St. Michael's Hospital, Toronto, ON, Canada; ^2^Department of Psychiatry, University of Toronto, Toronto, ON, Canada; ^3^Institute for Clinical Evaluatives Sciences, Toronto, ON, Canada; ^4^Applied Health Research Centre, Li Ka Shing Knowledge Institute, St. Michael's Hospital, Toronto, ON, Canada; ^5^Dalla Lana School of Public Health, University of Toronto, Toronto, ON, Canada; ^6^Division of General Internal Medicine, Department of Medicine, University of Toronto, Toronto, ON, Canada; ^7^Slaight Centre for Youth in Transition, Centre for Addiction and Mental Health, Toronto, ON, Canada; ^8^Institute for Health Policy Management and Evaluation, University of Toronto, Toronto, ON, Canada; ^9^Centre for Addiction and Mental Health, Toronto, ON, Canada

**Keywords:** empowerment, service engagement, homelessness, mental illness, recovery education

## Abstract

**Objective:** Grounded in principles of adult education, Recovery Education Centres (RECs) hold promise in promoting recovery for adults with mental health challenges, but research on recovery outcomes for hard-to-reach populations participating in RECs is scant. This quasi-experimental study compares 12-month recovery outcomes of adults with histories of homelessness and mental health challenges enrolled in a REC, to those of participants of other community services for this population.

**Methods:** This pre-post quasi-experimental study compared participants enrolled in a REC for people with histories of homelessness and mental health challenges (*n* = 92) to an age-and-gender frequency matched control group participating in usual services (*n* = 92) for this population in Toronto, Ontario. Changes from program enrollment to 12 months in personal empowerment (primary outcome), disease specific quality of life, recovery, health status, health related quality of life, and mastery were assessed. *Post-hoc* analyses compared subgroups with 1–13 h (*n* = 37) and 14+ h (*n* = 37) of REC participation during the study period to the control group. Linear mixed models estimated mean changes and differences in mean changes and 95% confidence intervals.

**Results:** Mean change in perceived empowerment from program enrollment to 12 months in the intervention group [0.10 (95% CI: 0.04, 0.15)] was not significantly different from the control group [0.05 (−0.01, 0.11)], mean difference, 0.05 [(−0.03, 0.13), *P* = 0.25]. In the *post-hoc* analysis, the mean change in perceived empowerment for the intervention subgroup with 14+ h of REC participation [0.18 (0.10, 0.26)] was significantly different than in the control group [0.05 (−0.01, 0.11)] mean difference, 0.13 [(0.03, 0.23), *P* < 0.01]. Mean change in mastery was also significantly different for the intervention subgroup with 14+ h of REC participation [2.03 (1.04, 3.02)] vs. controls [0.60 (−0.15, 1.35)], mean difference, 1.43 [(0.19, 2.66), *P* = 0.02]. There were no significant differences in other outcomes.

**Conclusion:** With sufficient hours of participation, recovery education may be a helpful adjunct to health and social services for adults with mental health challenges transitioning from homelessness.

## Introduction

Recovery has become the dominant paradigm in mental health in many countries (1). In the recovery context, individuals strive to reach their full potential and achieve satisfying, hopeful, contributing lives, and despite the limitations of mental illness (2).

Recovery Education Centres (RECs), offering education and role modelling in a judgment free non-clinical environment, are a promising approach to enhancing recovery outcomes for adults experiencing mental health challenges (1, 3, 4). First appearing in the United States in the 1990s, RECs have since been implemented in more than 20 countries, including the United Kingdom, the United States, Canada, Australia, New Zealand, and Europe (5, 6).

In qualitative studies, RECs have been found to support recovery outcomes at the personal, interpersonal and social levels, including perceived improvements in student connectedness with professionals (7) and student self-understanding and self-confidence (8). Longitudinal non-experimental quantitative studies have similarly identified benefits of participation, including greater participant knowledge and self-awareness, hopefulness, and mental wellbeing (9, 10), as well as reductions in service utilisation (11, 12). To our knowledge, only one published quantitative study of the impact of RECs to date has had a control group; this quasi-experimental evaluation reported short-term increases in participants' feelings of empowerment, self-efficacy, support, and affirmation (13).

Research on the effects of REC participation on disadvantaged populations, such as people transitioning from homelessness, has been scant (5, 14). This gap is salient given that this population faces multiple barriers to recovery, including high rates of complex health and social challenges, stigma, discrimination, and poor access to health services (15–19). RECs, promoting low barrier participation, choice, self-determination, social connection, personal growth, and role modeling through peers, could offer a helpful adjunct to services for this population (14, 20).

The primary objective of this quasi-experimental study was to compare the 12-month recovery outcomes of REC participants with histories of homelessness and mental health challenges, to those of a matched group of participants of other community services for this population in Toronto, Canada. A secondary objective was to examine, in a *post-hoc* analysis, the effect of the level of REC participation on these recovery outcomes.

For the primary analysis, we hypothesised that REC participants would experience greater increases in perceived empowerment (primary outcome), and quality of life, recovery, health status, and mastery (secondary outcomes) at 12 months, compared to a control group of participants engaged with other community services for this population.

## Methods

### Setting

The Supporting Transitions and Recovery Learning Centre (STAR) was launched in April 2014 at St. Michael's Hospital, an urban academic hospital in Toronto, Canada. Toronto is home to over 7,000 homeless people each night, with over 27,000 individuals using homeless shelters each year (21, 22). Toronto is also a service rich setting, in which physician, hospital services and community support services, including mental health services, are offered at no cost to individuals. In addition to health services for those experiencing homelessness, drop-in centres, social services and clubhouses offer recreational and vocational supports to this population.

### Study Design

This study used a pre-post quasi-experimental design. Although a randomised controlled trial (RCT) was initially considered, it was thought to compromise the choice options of a disadvantaged population and therefore not pursued at this time, given the centrality of choice in REC philosophy. A total of 92 individuals with mental health challenges and recent histories of homelessness were recruited to each of the intervention and control groups from January 2017 to July 2018 and followed for 12 months. Control group participants were frequency-matched to REC participants on age and self-identified gender (14). Data were collected at the time of study enrolment (baseline) and 6 and 12 months post baseline. To be eligible, intervention participants had to have registered in the REC during the recruitment period and met REC eligibility criteria: (i) experienced challenges with maintaining housing within the past 2 years and (ii) being over 16 years of age (14). Control group participants were individuals with no prior or current participation in the REC and were recruited from community agencies that provide non-clinical, low barrier social, recreational, and vocational supports for this population (e.g., drop-in centres, clubhouses, and community centres). Given the inclusive nature of Recovery Education Centres, diagnostic information about mental health or substance use challenges was not required. A detailed description of the study protocol has been published (14).

### Sample Size Calculation

A sample with at least 92 participants in each of the intervention and control groups provided 80% power to detect a medium effect size (Cohen *d* = 0.5) for the primary outcome, perceived empowerment, at 12 months, using a 2-sided *t*-test, assuming an attrition rate of 30% over the follow-up period. This was calculated using estimates from a quasi-experimental evaluation of a REC in Boston (13) that observed a difference in mean changes of 0.08 between the intervention and comparison groups from baseline to 12 months with an effect size of 0.41.

The study received Research Ethics Board approval by St. Michael's Hospital and the Centre for Addiction and Mental Health. Participation in the study was entirely voluntary. Prior to their baseline interview, individuals were asked for written informed consent. Capacity to consent was assessed by trained research staff who had experience working with this population. Specifically, together staff and participants reviewed a document with information about the study for participants to sign. When reviewing the consent form, interviewers were trained to regularly inquire if participants had questions. When a staff was uncertain about a participant's comprehension of the consent form, a consent checklist was used to confirm this comprehension. In addition, if a participant could not read or was visually impaired, another staff member was brought in to witness the consent portion of the interview and the participant's signature of the declaration of assistance.

Study participants received a $30 honorarium for attending each interview.

### Intervention

STAR is among few RECs worldwide with a mandate to support people transitioning out of homelessness, and to our knowledge, unique in North America. Individuals could self-refer to STAR or be referred from hospitals, primary care clinics, as well as local shelters, community organisations and other settings serving this population. They were required to register and develop individualised learning plans upon enrolment.

STAR classes and workshops were taught by peers with lived experience of housing instability and mental health challenges, as well as social and health service professionals. Classes focus on life skills related to transitioning from homelessness to housing and other topics such as health and wellness, vocational skills, leadership and community engagement, hobbies, and interests. While operated by a hospital, the central classroom and staff offices were located at a community centre and additional participation in learning opportunities is offered in partnership with other organisations (e.g., Employment Services Centre, Libraries).

### Outcomes

The primary outcome for this study was the change in self-reported personal empowerment from baseline to 12 months. Empowerment is a key dimension of recovery (23) and has been positively associated with perceived quality of life, self-esteem, and social support, and negatively associated with the severity of psychiatric symptoms (24). It was assessed using the Rogers Making Decisions Empowerment Scale (25). This measure has been used in other studies of individuals with mental health and addiction challenges (13), and has adequate psychometric properties, including internal consistency (alpha = 0.86) and factorial validity (26).

Secondary outcomes captured changes from baseline to 12 months in other domains inherent in the multidimensional view of recovery (see Table 1). These measures include:

- Quality of Life, measured with the Quality of Life Index 20 item (QoLi-20) (26).
- Quality of Recovery-Supporting Care, measured with INSPIRE (31, 32).
- Subjective Measures of Personal Recovery measured with QPR-15 (27).
- Health status measured with 12-Item Short Form Survey (SF-12) (28).
- Health Status (Health related quality of life) measured with EQ-5D-5L (29).
- Mastery, measured with Pearlin Mastery Scale (30).

**Table 1:** Outcomes, how they were measured, and missing data at each time point.

**Domain**	**Instrument**	**Missing data at each time point (%)**
**Primary outcome**		
Perceived empowerment	Rogers Making Decisions Empowerment Scale (25)	Baseline: 6.5%
		12 months: 5.4%
**Secondary outcomes**		
Disease specific quality of life	Quality of Life Index-20 Item (QoLi-20) (26)	Baseline: 0%
		12 months: 0%
Subjective measures of personal recovery	Questionnaire on the Process of Recovery (QPR-15) (27)	Baseline: 0%
		12 months: 1.4%
Health status	Short Form Survey (SF-12) (28)	Baseline: 0%
		12 months: 0.7%
Health status (health related quality of life)	EQ-5D-5L (29)	Baseline: 0%
		12 months: 0%
Mastery	Pearlin Mastery Scale (30)	Baseline: 0%
		12 months: 2.7%

For all scales, higher scores indicate greater recovery outcomes. Demographic variables that were collected at baseline were age, self-identified gender, ethnicity, employment, education, duration of longest period of homelessness, and housing stability over the past year, defined as the percentage of days stably housed. Housing stability was ascertained using the Modified Residential Time-Line Follow-Back Inventory that has high test–retest reliability and good concurrent validity in homeless populations (33).

### Analysis

Sociodemographic characteristics were compared for intervention and control group participants using the two-sample *t*-test or Fisher's exact test. To assess change in scores from baseline to 12 months, as per the protocol analysis (14), linear mixed models with random intercepts were applied to perform repeated measures analysis of perceived empowerment and secondary outcomes (all continuous). An exchangeable correlation structure was used. The model included the main fixed effects of group (intervention vs. comparison), time (12 months vs. baseline), and the intervention × month interaction, adjusting for participant's age at baseline, self-identified gender, ethnicity, education, longest period of homelessness, and housing stability over the past year (*a priori* analysis). For each outcome, model-estimated mean changes from baseline to 12 months follow up and 95% CIs were examined. Differences in mean changes, 95% CIs and *p*-values were calculated to compare the groups (34).

We conducted an “as-treated” *post-hoc* analysis to examine the effect of participation, as has been done in other studies on recovery services (35). This involved examining the effect of the number of hours of REC participation, rather than group membership. There is no precedent for the minimum “dose” of REC participation (i.e., number of sessions attended, or amount of time spent attending) needed to support recovery in RECs. As such, we consulted a review of 23 studies on similar interventions (i.e., peer led group sessions for participants with serious mental illness) and with similar designs to the present evaluation (longitudinal design, recovery outcomes of interest) (36). Of 23 studies, seven reported the mean number of sessions that participants attended [mean = 6.5 sessions or 13 h (6.5 × 2 h)].

To examine the effect of varying levels of participation on changes in primary and secondary outcomes from baseline to 12 months, we created three intervention subgroups based on hours of REC participation: REC registrants with no hours of participation, and therefore no exposure to the intervention (*n* = 18); (ii) REC registrants with 1–13 h of REC participation (*n* = 37), and (iii) REC registrants with 14 or more REC hours of participation during the 12 month follow up period (*n* = 37). The mixed effects model described above was applied again for this analysis, with the main fixed effect of group replaced with the hours of participation variable (0 h, 1–13 h, 14 or more hours, vs. control group).

Most scales had little missing data, or none (see Table 1). All statistical tests were two-sided, and a value of 0.05 or less indicated statistical significance. R software (version 3.4.1) was used for all analyses (37).

## Results

At baseline, across both groups (*n* = 184), participants had a median age of 42.5 years (IQR: 28.15–53.20). The majority were unemployed (76.1%, Table 2A), female (51.1%) non-white (52.2%), and not stably housed over the previous year (74.0%). Compared to the control group, the intervention group included a significantly lower proportion of participants who did not complete high school (19.6 vs. 43.5%, *p* < 0.001), and had shorter durations of homelessness [median, in years (IQR): 0.84 (0.33–2.00) vs. 1.00 (0.54–4.00), *p* < 0.05].

**Table 2A:** Sociodemographic characteristics for the intervention group and control groups at baseline.

**Characteristic**		**All** **(***N*** = 184)**	**Intervention** **(***N*** = 92)**	**Control** **(***N*** = 92)**	***P*** -value^*^
# Hours of REC participation during the study period	Median (IQR)	0.00 (0.00–6.00)	6.00 (2.00–42.00)	0.0 (0.0–0.0)	
Age at baseline	Median (IQR)	42.50 (28.15–53.20)	40.29 (30.38–53.20)	44.14 (25.59–53.20)	0.55
Longest period of homelessness (Years)	Median (IQR)	1.00 (0.42–3.00)	0.84 (0.33–2.00)	1.00 (0.54–4.00)	<0.05
		***n*** **(%)**	***n*** **(%)**	***n*** **(%)**	
Self-identified gender	Male	88 (47.8)	45 (48.9)	43 (46.7)	0.53
Ethnicity	White	87 (47.8)	49 (53.3)	38 (42.2)	0.14
	Other	95 (52.2)	43 (46.7)	52 (57.8)	
Education level	Did not finish high school (or less)	58 (31.5)	18 (19.6)	40 (43.5)	<0.01
	Attended or completed business, trade, technical or high school	76 (41.3)	38 (41.3)	38 (41.3)	
	Attended University or Post-Secondary Grad	50 (27.17)	36 (39.1)	14 (15.2)	
Employment status	Unemployed	140 (76.1)	75 (81.5)	65 (70.7)	0.12
Housing status (past year)	Not stably housed	131 (74.0)	68 (76.4)	63 (71.6)	0.50

* *Two-sample t-tests and Fisher's exact tests were used*.

During the 12-month follow up period, 40.2% of the intervention group (*n* = 37) had 1–13 h of REC participation (median: 4.00 h, IQR: 2.00–6.00). Another 40.2% (*n* = 37) had 14 or more hours of REC participation (median: 54.00 h, IQR: 31.00–101.00, Table 2B). Relative to the control group, individuals with 14 or more REC participation hours were older [median: 48.10 years (IQR: 33.61–54.35)] vs. 44.14 years (IQR: 25.59–53.24), more likely to be white (73.0 vs. 42.2%), unemployed (86.5 vs. 70.7%), had more than high school education (94.6 vs. 59.1%), and had shorter duration of lifetime homelessness [median: 0.76 (IQR: 0.33–1.50)] vs. 1.00 (0.54–4.00) year).

**Table 2B:** Sociodemographic characteristics for the total sample and for the intervention subgroups divided by number of hours of REC participation over the 12 month study period.

					**Intervention subgroup**		
		**All participants**	**Control group**	**Intervention group**	**0 h**	**1–13 h**	**14+ h**
		**(***N*** = 184)**	**(***N*** = 92)**	**(***N*** = 92)**	**(***N*** = 18)**	**(***N*** = 37)**	**(***N*** = 37)**
		**Median (IQR)**					
# Hours of REC participation during the study period up	Median (IQR)	0.00 (0.00–6.00)	0.00 (0.00–0.00)	6.00 (2.00–42.00)	0.00 (0.00–0.00)	4.00 (2.00–6.00)	54.00 (31.00–101.00)
Age at baseline (Years)	Median (IQR)	42.50 (28.15–53.24)	44.14 (25.59–53.24)	40.29 (30.38–53.20)	34.73 (30.78–50.19)	40.34 (29.30–52.82)	48.10 (33.61–54.35)
Longest single period of homelessness (Years)	Median (IQR)	1.00 (0.42–3.00)	1.00 (0.54–4.00)	0.84 (0.33–2.00)	0.96 (0.42–3.75)	1.00 (0.31–3.00)	0.76 (0.33–1.50)
		***n*** **(%)**					
Self-identified gender	Male	88 (47.8)	43 (46.7)	45 (48.9)	9 (50.0)	19 (51.4)	17 (45.6)
Ethnicity	White	87 (47.8)	38 (42.2)	49 (53.3)	8 (44.4)	14 (37.8)	27 (73.0)
Education level	Did not finish high school (or less)	58 (31.5)	40 (43.5)	18 (19.6)	5 (27.8)	11 (29.7)	2 (5.4)
	Attended business, trade, technical or high school	76 (41.3)	38 (41.3)	38 (41.3)	7 (38.9)	11 (29.7)	20 (54.1)
	Attended University or Post-Secondary Grad	50 (27.2)	14 (15.2)	36 (39.1)	6 (33.3)	15 (40.5)	15 (40.5)
Employment status	Unemployed	140 (76.1)	65 (70.7)	75 (81.5)	13 (72.2)	30 (81.1)	32 (86.5)
Housing status (past year)	Not stably housed	131 (74.0)	63 (71.6)	68 (76.4)	14 (82.4)	28 (77.8)	26 (72.2)

### A Priori Analysis

#### Primary Outcome

Mean change (Table 3) in perceived empowerment score from baseline to 12 months among intervention group participants [0.10 (95% CI: 0.04, 0.15)] was not statistically different from control group participants (0.05 (95% CI: −0.01, 0.11), mean difference, 0.05 (−0.03, 0.13), *p* = 0.25). For mean scores and standard deviations at baseline and 12 months (see Appendix A).

**Table 3:** Model-estimated mean changes and 95% CIs, and difference in mean changes, 95% CIs and *p*-values from baseline to 12 months follow up for intervention and control group participants (a priori analysis).

	**Mean change (95% CI) from baseline to 12 months**		**Difference in mean changes (95% CI)**, ***P*****-values**	
	**Intervention** **(***n*** = 92)**	**Control** **(***n*** = 92)**	**Intervention vs. control**	
	**Mean change (95% CI)**	**Mean change (95% CI)**	**Mean change (95% CI)**	***P*** **-values**
Perceived empowerment	0.10 (0.04, 0.15)	0.05 (−0.01, 0.11)	0.05 (−0.03, 0.13)	0.25
Disease specific quality of life	7.14 (3.00, 11.27)	6.00 (1.63, 10.36)	1.15 (−4.87, 7.17)	0.71
Recovery	1.89 (−0.26, 4.04)	2.06 (−0.23, 4.36)	−0.17 (−3.32, 2.98)	0.92
Physical health status	−0.23 (−2.22, 1.75)	0.22 (−1.87, 2.32)	−0.44 (−3.33, 2.44)	0.76
Mental health status	4.05 (1.55, 6.54)	2.14 (−0.49, 4.77)	1.91 (−1.72, 5.53)	0.30
Health-related quality of life	8.46 (3.58, 13.33)	4.37 (−0.76, 9.51)	4.08 (−3.00, 11.16)	0.26
Mastery	0.56 (−0.14, 1.25)	0.60 (−0.15, 1.35)	−0.05 (−1.07, 0.98)	0.93

#### Secondary Outcomes

Mean changes from baseline did not differ significantly between the intervention and control groups at 12 months for disease specific quality of life (QoLi-20)—Intervention: 7.14 (3.00, 11.27), Control: 6.00 (1.63, 10.36), mean difference, 1.15 [(−4.87, 7.17), *p* = 0.71]; Mental health status (MCS)—Intervention: 4.05 (1.55–6.54), Control: 2.14 (−0.49 to 4.77), mean difference, 1.91 [(−1.72, 5.53), *p* = 0.30]; Health related quality of life (EQ-5D)-Intervention: 8.46 (3.58, 13.33), Control: 4.37 (−0.76, 9.51), mean difference, 4.08 [(−3.00, 11.16), *p* = 0.26, Table 3].

### *Post-hoc* Analysis

#### Primary Outcome

The mean changes for perceived empowerment from baseline to 12 months for the 14 or more REC hours participant group were statistically different from the control group {Mean differences and 95% CI: [0.18 (0.10, 0.26) vs. 0.05 (−0.01, 0.11)] difference in mean changes <0.01, Table 4}.

**Table 4:** Model-estimated mean changes and 95% CIs, and difference in mean changes, 95% CIs and *p*-values from baseline to 12 months follow up for individuals with 0, 1–13, and 14+ REC participation hours vs. the control group (*post-hoc* analysis).

	**Mean change (95% CI) from baseline, 12 months**	**Difference in mean changes (95% CI)**, ***P*****-values**	
	**Control** **(***n*** = 92)**		
	**Mean change (95% CI)**		
Perceived empowerment	0.05 (−0.01, 0.11)		
Disease specific quality of life	6.00 (1.63, 10.36)		
Recovery	2.06 (−0.23, 4.36)		
Physical health status	0.22 (−1.87, 2.32)		
Mental health status	2.14 (−0.49, 4.77)		
Health-related quality of life	4.37 (−0.76, 9.51)		
Mastery	0.60 (−0.15, 1.35)		
	**0 REC hours** **(***n*** = 18)**	**0 REC hours group vs. control group**		
	**Mean change (95% CI)**	**Mean change (95% CI)**	***P*** **-values**
Perceived empowerment	0.08 (−0.06, 0.21)	0.03 (−0.12, 0.17)	0.69
Disease specific quality of life	10.68 (0.64, 20.72)	4.68 (−6.28, 15.64)	0.40
Recovery	−0.88 (−6.06, 4.29)	−2.95 (−8.62, 2.71)	0.31
Physical health status	3.36 (−1.43, 8.15)	3.14 (−2.09, 8.36)	0.24
Mental health status	0.82 (−5.18, 6.83)	−1.32 (−7.88, 5.24)	0.69
Health-related quality of life	12.40 (0.69, 24.12)	8.03 (−4.77, 20.83)	0.22
Mastery	−0.75 (−2.39, 0.89)	−1.35 (−3.15, 0.44)	0.14
	**1–13 REC hours (*****n*** **=** **37)**	**1–13 REC hours group vs. control group**		
	**Mean change (95% CI)**	**Mean change (95% CI)**	***P*** **-values**
Perceived empowerment	−0.01 (−0.10, 0.08)	−0.06 (−0.17, 0.05)	0.31
Disease specific quality of life	5.60 (−1.38, 12.58)	−0.40 (−8.65, 7.85)	0.92
Recovery	0.17 (−3.43, 3.77)	−1.90 (−6.17, 2.37)	0.38
Physical health status	−0.34 (−3.67, 2.98)	−0.56 (−4.50, 3.37)	0.78
Mental health status	2.89 (−1.29, 7.07)	0.75 (−4.20, 5.69)	0.77
Health-related quality of life	5.86 (−2.32, 14.03)	1.48 (−8.18, 11.15)	0.76
Mastery	−0.71 (−1.85, 0.43)	−1.31 (−2.67, 0.04)	0.06
	**14+** **REC hours** **(***n*** = 37)**	**14+ REC hours group vs. control group**		
	**Mean change (95% CI)**	**Mean change (95% CI)**	***P*** **-values**
Perceived empowerment	0.18 (0.10, 0.26)	0.13 (0.03, 0.23)	<0.01
Disease specific quality of life	7.10 (1.02, 13.18)	1.10 (−6.41, 8.61)	0.77
Recovery	4.29 (1.15, 7.42)	2.22 (−1.67, 6.10)	0.26
Physical health status	−1.47 (−4.36, 1.43)	−1.69 (−5.26, 1.89)	0.35
Mental health status	6.25 (2.60, 9.91)	4.11 (−0.39, 8.61)	0.07
Health-related quality of life	8.89 (1.71, 16.06)	4.51 (−4.32, 13.35)	0.32
Mastery	2.03 (1.04, 3.02)	1.43 (0.19, 2.66)	0.02

#### Secondary Outcomes

The mean changes for mastery from baseline to 12 months for the 14 or more REC hours participant group were statistically different compared to the control group [2.03 (1.04, 3.02) vs. 0.60 (−0.15, 1.35), mean difference, 1.43 (0.19, 2.66), *P* = 0.02]. For other outcomes, mean differences did not differ significantly between the 14 or more REC hours group and the control group at 12 months (Table 4).

Mean changes from baseline to 12 months for the group with 1–13 REC participation hours were not statistically different from those of the control group (Table 4).

## Discussion

This study evaluated the impact of REC membership and participation on key recovery outcomes for people with mental health challenges transitioning from homelessness in Toronto, Canada. This is only the second controlled longitudinal study of REC outcomes, following Dunn et al. (13), and is the first study examining outcomes for a population facing multiple barriers to mental health recovery. Compared to an age-and-gender frequency matched control group, REC participants did not experience statistically significant differences in any of the outcomes examined. However, both groups experienced improvement in most outcomes over 12 months.

Improvements in recovery outcomes among REC participants were also noted in qualitative findings from this evaluation (38), highlighting perceived improvements in health and well-being, self-esteem and confidence, interpersonal skills, and goal orientation (4).

Among the 92 REC study participants, 18 participants (20.0% of the intervention group) had no hours of participation in the REC. This is similar to the proportion of program registrants with no participation observed in other studies on recovery-oriented peer services, including recovery colleges. For example, in a study on a United Kingdom based REC, 29% of students who registered for courses never attended (11). Similarly, a study of veterans with mental health challenges who were using recovery-oriented peer support services found that 26% did not participate in learning opportunities (35). Finally, in a study of patients who were assigned a peer mentor after psychiatric hospitalisation, 34% had no contact with their mentor (39). Although a study on engagement with peer led services among previously homeless veterans with co-occurring mental illness and histories of substance use (40) did not report the proportion of participants with no attendance, it was similarly noted that participants had about one contact with the program per month, less than the intended once per week.

The *post-hoc* analysis highlighted that, after 12 months, those with 14 or more hours of REC participation had significantly greater improvements in perceived empowerment and mastery scores than control group participants. These finding suggest that a minimum level of REC participation may be needed to achieve key recovery outcomes. These findings support and are supported by a recent Australian study (41), in which attending a higher percentage of REC courses was associated with goal achievement. The *post-hoc* analysis further identified that socio-demographic characteristics were associated with the extent of participation. Specifically, participation was lower in people who were younger, non-white, unstably housed, employed, and had fewer years of education. As program participation is one of several pathways to improved recovery outcomes, our findings highlight a current gap in knowledge, as little evidence exists on the intensity of supports needed in REC and other recovery focused services for disadvantaged populations (40).

Future research should examine barriers to participating in RECs for individuals who experience multiple forms of disadvantage, such as being racialized, and having low levels of education. The social location of multiply disadvantaged groups can result in multiple barriers to accessing or engaging with available resources, producing differential effects on health and well-being (42). Finally, future research should be used to gain a more nuanced understanding of how RECs compare to other programs in terms of costs and on the reasons for limited or no subsequent engagement with RECs and other low barrier services after program enrollment.

The only other longitudinal evaluation of a REC with a comparison group (13) was conducted in Boston, USA in the 2000s and similarly examined the effects of REC participation on empowerment and other outcomes for individuals with mental health challenges relative to users of services-as-usual. There were significant increases in perceived empowerment and recovery attitudes for the intervention group relative to the comparison group, that experienced deterioration over time. Key differences from the present study are that the REC was not targeted to homeless individuals, and that a minimum of 36 h of participation was required to be eligible for study enrolment.

### Limitations and Strengths

Although a randomised controlled trial (RCT) could have yielded stronger findings, pragmatic considerations advised against an RCT design at this time (13, 14). While the *post-hoc* analysis showed that REC participation for 14+ h was associated with improvements in perceived empowerment and mastery, it is notable that the intervention subgroup with 14+ h of participation in the REC was older, more likely to be white, to have attended University or post-secondary education, and less likely to be employed than the control group. Although multivariable models were adjusted for participant age, self-identified gender, ethnicity, education, longest period of homelessness, and housing stability over the past year, it is possible that greater participation in RECs could be associated with unmeasured individual characteristics that result in greater increases in self-reported empowerment. Additionally, the dose threshold of 14 h for recovery education was determined by a review of studies that examined the published the number of sessions needed to support recovery. However, the mean number rather than median number of sessions was used so it is possible that the dose determination may have been overly skewed. An additional limitation is that the study, similar to other intervention studies (18, 42) did not capture additional services used by the intervention or control groups. This lack of information is particularly salient, as both the intervention and control groups experienced improvements over time. Another limitation is that in the field of patient-reported outcomes, such as empowerment, there are not standards established as to how to measure minimally important differences in outcomes (43).

Despite these limitations, this study has notable strengths compared to previous studies of REC outcomes, plagued by small sample sizes, and non-experimental designs. Importantly, the present study used rigorous quasi-experimental methods, focusing on a disadvantaged population experiencing multiple barriers to recovery, and included a control group of participants engaged with other community services. Another strength is the 12 month longitudinal follow-up period. In the review (36) of 23 longitudinal studies with similar outcomes cited earlier, only four had follow up periods of a year or more. Finally, grouping REC participants by hours of participation allowed for a more nuanced understanding of the potential impact of hours of participation on recovery outcomes.

## Conclusion

Engaging people transitioning out of homelessness in Recovery Education may be a helpful adjunct to other services and supports for this population, although a minimum “dose” of REC participation may be needed to observe improvements in recovery outcomes. Future research should examine the processes and mechanisms that promote participation and engagement with recovery education, and the impact on recovery outcomes.

## Data Availability Statement

The raw data supporting the conclusions of this article will be made available by the authors, without undue reservation.

## Ethics Statement

The studies involving human participants were reviewed and approved by St. Michael's Hospital and the Centre for Addiction and Mental Health. The patients/participants provided their written informed consent to participate in this study. Written informed consent was obtained from the individual(s) for the publication of any potentially identifiable images or data included in this article.

## Author Contributions

AD led manuscript drafting. VS was the study's Principal Investigator and supervised the drafting of this manuscript. RN and RW led the data analysis and participated in the editing of this manuscript. SH and NK participated in study design and implementation and the editing of this manuscript. All authors contributed to the article and approved the submitted version.

## Funding

This study was funded by the Canadian Institutes for Health Research. The STAR Learning Centre was funded by the Odette Fund for Homeless People, St. Michael's Hospital Foundation.

## Conflict of Interest

The authors declare that the research was conducted in the absence of any commercial or financial relationships that could be construed as a potential conflict of interest.

## Publisher's Note

All claims expressed in this article are solely those of the authors and do not necessarily represent those of their affiliated organizations, or those of the publisher, the editors and the reviewers. Any product that may be evaluated in this article, or claim that may be made by its manufacturer, is not guaranteed or endorsed by the publisher.

## Appendix

**Table A1:** Mean scores and standard deviations (SDs) on outcome measures at baseline and at 12 months follow-up for intervention and control group participants (*a priori* analysis).

	**Scores at baseline**		**Scores at 12 months**	
	**Intervention (*n* = 92)**	**Control (*n* = 92)**	**Intervention (*n* = 92)**	**Control (*n* = 92)**
	**Mean (SD)**	**Mean (SD)**	**Mean (SD)**	**Mean (SD)**
**Raw scores**				
Perceived empowerment	2.94 (0.23)	2.84 (0.28)	3.02 (0.29)	2.89 (0.28)
Disease Specific Quality of Life	83.60 (22.23)	80.81 (17.59)	90.17 (21.90)	86.48 (20.73)
Recovery	41.50 (10.35)	39.50 (9.73)	43.30 (9.18)	40.39 (11.01)
Physical health status	48.58 (10.26)	45.93 (11.25)	49.13 (9.83)	44.95 (10.82)
Mental health status	38.87 (10.80)	38.44 (11.62)	42.45 (12.59)	40.83 (11.09)
Health-related quality of life	63.83 (18.33)	61.54 (20.18)	71.62 (19.60)	65.83 (22.60)
Mastery	19.65 (3.63)	18.90 (3.43)	20.24 (3.39)	19.40 (3.04)
	**Mean (95% CI)**	**Mean (95% CI)**	**Mean (95% CI)**	**Mean (95% CI)**
**Adjusted scores derived from complete models**				
Perceived empowerment	2.96 (0.09)	2.89 (0.09)	3.05 (0.09)	2.93 (0.09)
Disease Specific Quality of Life	82.71 (6.49)	79.70 (6.27)	89.84 (6.53)	85.69 (6.33)
Recovery	41.38 (3.47)	39.70 (3.36)	43.27 (3.49)	41.76 (3.39)
Physical health status	40.82 (3.25)	39.72 (3.14)	40.59 (3.27)	39.94 (3.17)
Mental health status	41.50 (3.56)	41.44 (3.45)	45.55 (3.59)	43.57 (3.48)
Health-related quality of life	63.13 (6.06)	61.28 (5.86)	71.59 (6.12)	65.65 (5.95)
Mastery	19.58 (1.09)	18.78 (1.05)	20.14 (1.09)	19.38 (1.06)
